# Deriving the Types and Characteristics of Lost Children in South Korea Using the Sequential Association Rule

**DOI:** 10.3390/bs13050393

**Published:** 2023-05-09

**Authors:** Soyoung Choi

**Affiliations:** Institute of Construction and Environmental Engineering, Seoul National University, Seoul 08826, Republic of Korea; y5087@snu.ac.kr; Tel.: +82-2-880-8869

**Keywords:** lost child, missing child, cause of lost child, type of lost child, process of lost child

## Abstract

The studies that examine the characteristics of lost children and identify the types and processes of children being lost are insufficient. Therefore, this study aimed to establish the fundamental types and characteristics of lost children and develop a plan for their prevention. First, the common patterns of lost children were derived via the “sequential association rule” using the lost child case data from the previous studies. Next, the lost child types were derived by examining the patterns of lost children, focusing on the situation (conditions) before the child was lost and the causes. Additionally, a series of processes leading to children getting lost and being reunited with their guardians, according to the lost child type, were systematized. Finally, the causes and characteristics of lost children were derived for each type. The following types of lost children were derived: type I—when a child unexpectedly breaks away from the guardian and becomes lost; type II—when a child leaves after obtaining the guardian’s permission but fails to find their way back to their guardian; and type III—when a guardian and child are separated by the operation of transportation. This study’s findings can assist in the development of environmental design guidelines to prevent children from getting lost.

## 1. Introduction

The reasons for missing children are generally classified into abduction, abandonment, runaways, injured children (due to accidents), and lost children, among others [1,2,3]. According to a survey of 500 elementary students in Korea, 22.1% had gotten lost and 1.0% had been kidnapped [4]. As such, cases of lost children occur frequently, and this number is significantly higher than that of other disappearances. However, the importance of lost child studies is often underestimated because most lost children are usually found within 48 h [5]. In other words, the fundamental studies focusing on lost children are insufficient, as most studies focus on comprehensive missing children (e.g., Sidebottom et al. [6]; Boulton et al. [7]). Most national surveys on lost children in developed countries focus on identifying the current status of lost children based on statistics, rather than conducting in-depth analyses and considerations (e.g., [2,3,8]). Additionally, the research on lost children mainly focuses on follow-up measures to find the children based on smart devices or artificial intelligence (AI) systems (e.g., [9,10,11]). There is a lack of basic theoretical studies that examine the essential characteristics of how children are lost. Few studies systematically and logically identify the types and processes of children becoming lost. Since a disappearance due to getting lost entails various problems, including psychological and economic damage to children and their families, it is necessary to prevent these occurrences in advance rather than detect them shortly after the children disappear [5]. Additionally, there are few studies on missing children based on the academic exploration of child development and missing behavior [12], and only a few studies have examined the factors that influence the spatial behavior of missing people [13]. In this regard, this fundamental study aims to develop an environmental plan to prevent children from getting lost by establishing the types and characteristics of lost children based on functional spaces and behavior. The types of lost children were established by examining the processes and characteristics of their environment and the behavior of the guardians and children, which cause lost children in each space. This study utilized the following method. First, common patterns of lost children were derived by conducting a “sequential association rule” using the lost child case data from the previous studies. Next, the types of lost children were derived by examining their patterns, focusing on the situation (conditions) before the child got lost as well as the causes. Additionally, a series of processes leading to children getting lost and being reunited with their guardians, according to the type of lost child, were systematized. Finally, the causes and characteristics of lost children were derived for each type. As a fundamental study on lost children, this study can help the childcare and architecture fields by establishing environmental design guidelines to prevent lost children.

## 2. Theoretical Study

### 2.1. Causes of Lost Children

A “lost child” refers to a child who has lost their way or is unable to return home on their own due to the lack of geographic discernment or cognitive ability after separation from their guardian [14]. Although there are various causes that lead to a child getting lost, it may be said that a lost child is mainly caused by the negligence of guardians, characteristics of children, and characteristics of the environment [15].

According to a lost children case study by Choi et al. [16], among 202 cases, 87.6% were affected by guardian-related causes, 81.7% by child-related causes, and 61.4% by environmental causes. This result suggests that a child getting lost is not solely affected by one cause (8.5%) but rather a combination of various causes (91.5%) originating from the guardian, child, and environment. Since both the guardian and the child causes contributed to 34.2% of child loss cases, and the combined causes of the guardian, child, and the environment accounted for 39.1%, this suggests that the environmental characteristics acted as the catalyst by inducing child loss and making it more difficult for guardians to find their child [16]. The causes of lost children revealed in this previous study are listed in Table 1.

### 2.2. Characteristics of Child Development That Cause Lost Children

Considering the characteristics of child development, the following aspects may lead to lost children, as children suddenly leave their guardians or spaces or fail to find their way. First, when children reach the age of two, they can move more skillfully and have a strong desire for movement. Consequently, they want to move around without remaining in a limited space [17,18]. The development of their locomotion capacity enables them to explore their surroundings more extensively than during infancy, making them more independent [17]. Accordingly, children at this age may become lost as they leave the space due to their excessive activity and independence. The average height of 3-year-old children, who are most likely to become lost, is about 1 m [19]. They are, thus, short and have low eye levels. In addition, regarding viewing angles, adults can see in a vertical direction of 120° and horizontal direction of 150° through both eyes. In contrast, young children can only see in a vertical direction of 70° and horizontal direction of 90° [20]. Therefore, they may not be fully aware of their surroundings and may not be able to quickly locate their guardians. At this moment, children may recognize themselves as lost and move in search of their guardians. Children can also be lost due to visual obstacles preventing their guardians from finding them. Furthermore, a sign system that does not account for the limitations of a child’s eye level and viewing angle may hinder their wayfinding by making it difficult for them to locate the sign.

Ages one to three comprise the period when children’s autonomy is established, and they express their will to do things alone [18]. Ages three to five are when their initiative is formed, and they are curious and enjoy trying new things [21]. Children may lose their way while walking alone or moving to a specific place due to this autonomy and initiative. As the thought patterns of children aged two to three years are centered on themselves due to egocentrism, they may become lost as they move alone without considering their guardians. Additionally, as attention abilities increase around the age of five or six, children younger than this have a short attention span and are impulsive [22,23]. As such, the younger the child, the lower the concentration regarding their attention. If guardians overlook this and ask children to wait for a while and then leave the space, it can result in the child being lost. At the age of three, children’s memory develops rapidly [21]. Thus, children of three years and older may become lost due to recalling interesting elements or places in their memories and, consequently, moving to the spaces alone. Moreover, around the age of nine to twelve, children are able to perceive space as a perfect single coordinate system [24]. In other words, children under the age of nine may become lost as they fail to find their way because their sense of direction, distance, and relative position is incomplete.

Similarly, children can become lost due to various developmental characteristics. However, if guardians control children’s behavior to prevent them from being lost, children may not be able to promptly achieve their appropriate physical, emotional, and cognitive development. Therefore, guardians need to support children’s activities without restricting their development. In contrast, child-related spaces are required to create environments where guardians can easily observe children and avoid losing them.

### 2.3. Methods

In this study, data mining was conducted to classify the lost child case data into several patterns and derive the types of lost children. Data mining techniques involve a process of identifying and classifying data patterns and rules so that useful information can be obtained from large datasets to make decisions or judgments [25,26,27,28]. For example, by identifying the different attributes (structure, height, or unit) of each building in the datasets, the buildings can be easily categorized into different types (based on the occupancy or type of construction) [25]. Patterns in the database are described by relationships between the attributes [27]. In data mining, a classification task involves classifying each item in a dataset into classes or groups [25,26,28]. The representative techniques of data mining include clustering (cluster analysis), decision trees, and association rule/sequential patterns (sequential association rule) [25,26,27,28].

Clustering is the unsupervised classification of patterns (observations, data items, or feature vectors) into groups (clusters) [29]. It involves the process of identifying natural groupings or clusters within multidimensional data based on a similarity measure, such as the Euclidean distance [29,30,31]. Clustering algorithms include hierarchical and partitional (non-hierarchical) clustering. Hierarchical clustering is a method for continuously reducing the number of clusters while merging them with the highest similarity or close distance among several clusters (e.g., single, complete, and average linkage, etc.). Partitioning clustering is based on specifying an initial number of groups and iteratively reallocating the objects among the groups to achieve convergence. Thus, clustering typically determines all the clusters at once (e.g., K-means algorithm) [32].

A decision tree is an inverse tree-shaped hierarchical process in which the root node is split into sub-branches and the leaf node contains a class label [26,33]. A decision tree starts with a simple question that has two answers. Every answer drives an extra question to support the classification or identification of the data. Thus, a categorization or prediction could be made based on each answer [25]. The disadvantage of a decision tree is that it can be subject to overfitting and underfitting, particularly when using a small dataset [27,33]. Another problem is that a strong correlation between the different input variables may result in a selection of variables that improve the model statistics but are not causally related to the outcome of interest [33]. Therefore, one must be cautious when using decision tree models to develop causal hypotheses [33].

The association rule devised by Agrawal identifies relevant rules and patterns between sets of items hidden in the data using unsupervised learning in large databases [34]. This technique, used to determine the correlation between two or more item groups, derives a rule in the ‘If–Then’ format. This means that “if A occurs, then B happens”, expressed as “A→B”. The sequential association rule is a technique for identifying the correlation between events according to the passage of time by adding the concept of time to the association rule [35]. While it is similar to the association rule, it is different in that the latter is analyzed by focusing on the events that co-occurred. In contrast, the sequential association rule is analyzed by concentrating on the events according to the time/order. Thus, the time information variable must exist in the dataset of the sequential association rule. This analysis technique reflects the concept of time, making it useful when analyzing time-series data in which the order of time is important, such as the order of purchases. However, since the association rule does not reflect the time/order, there is a disadvantage in that several unnecessary patterns or rules that do not match the actual situation may be derived.

In this study, in terms of classifying the types based on the various causes in the flow of time when children get lost, the association rule that identified the associations in the form of conditions and reactions, as well as causes and consequences (if–then), was more appropriate than the previous methods. In addition, since time-series data were used, the sequential association rule was used as a data mining method. Regarding the instance of a lost child, when the rule—“guardian’s absence” and “child’s leaving” are related—is derived from the analysis of association rules, rules that do not make sense, such as “if a child leaves, the guardian goes off”, may be derived. Nonetheless, in practice, the rule that a child has become lost because “the child leaves after the guardian goes off” is reasonable. By using the sequential association rule, this problem can be overcome. It is not that various situations and causes simultaneously affect the occurrence of lost children, but rather that children can get lost over time through a series of processes, from situations in which a guardian and a child are together to the child leaving and returning to the guardian. Based on this, this study attempts to derive the types of situations that cause lost children using the sequential association rule.

The indicators for evaluating the usefulness of the rules in sequential association include the support, confidence, and lift, similar to the association rule. Support refers to the ratio of transactions containing item A and item B among all the transactions. Confidence refers to the ratio of the transactions, including item A and item B, among the transactions involving item A. Lift refers to the ratio of the increase in the probability that item B occurs in a transaction in which item A happens compared to the transaction in which item B occurs. Lift is an index for evaluating whether item A and item B occurred simultaneously by chance. If item A and item B are not related to each other, that is, if they are independent, lift becomes 1. If the lift is between 0 and 1, the two items have a negative correlation with each other, and if it is greater than 1, the two items have a positive correlation with each other, which can be interpreted as a rule with a significant correlation rather than a coincidence. These rules can be expressed as the following equations.

$$Support=P\left(A\cap B\right)=\frac{A\cap B}{Total}+\frac{Transactions\;containing\;both\;A\;and\;B}{Total\;number\;of\;transactions}$$

$$Confidence=\frac{P\left(A\cap B\right)}{P\left(A\right)}=\frac{Transactions\;containing\;both\;A\;and\;B}{Transactions\;containing\;A}=\frac{Support}{P\left(A\right)}$$

$$Lift=\frac{P\left(A\cap B\right)}{P\left(A\right)\times P\left(B\right)}=\frac{Transactions\;containing\;both\;A\;and\;B}{Transactions\;containing\;A\times Transactions\;containing\;B}=\frac{Confidence}{P\left(B\right)}$$

**Figure 1:**
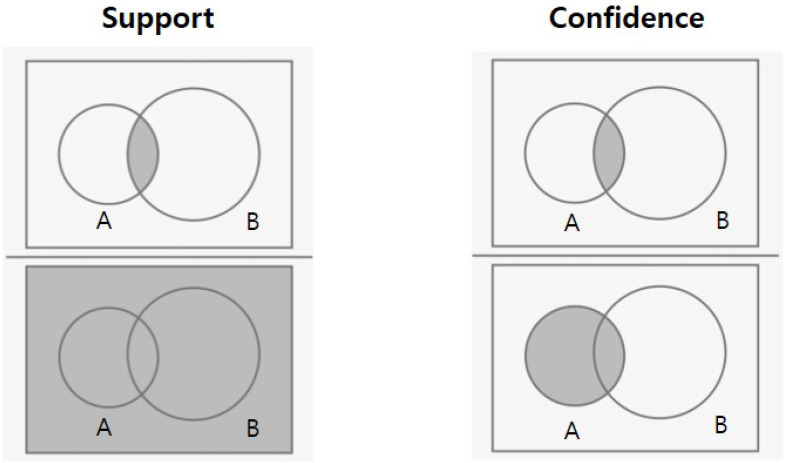
Concepts of the support and confidence.

Among these three indicators, the support is an indicator of how representative the rule is. However, regarding a rule with a low frequency, the support value appears relatively low. Even if the correlation between item A and item B is significant, the support value may be small. To compensate for these blind spots, the confidence index, a criterion for evaluating the accuracy of a rule, can be used. In addition, meaningless rules can be removed, and they can be derived using the lift, an index indicating the coincidence and intensity of the rules. A generalized rule among the numerous rules can be derived based on these three indicators. The concepts of the support and confidence are illustrated in Figure 1.

## 3. Sequential Association Rule for Lost Children

### 3.1. Analysis Method

**Figure 2:**
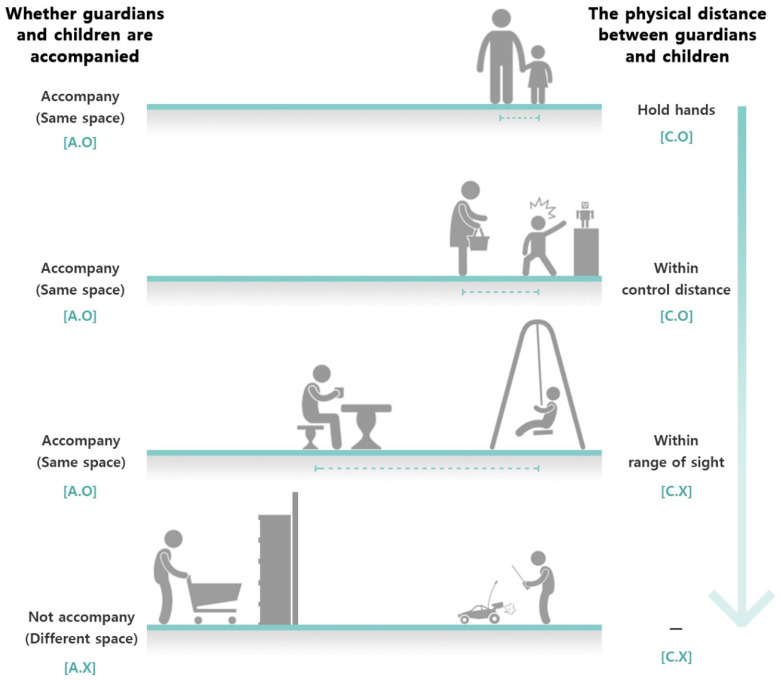
Criteria for whether children are accompanied by guardians and their physical distance.

In this study, the types that caused lost children were derived based on the existing 202 lost children cases studied by Choi et al. [16]. This study analyzed the sequential association rules based on the following criteria: lost children are not simply caused by a combination of causes, there are situations (conditions) that cause lost children before the cause has an effect. The first prerequisite was whether the guardian and the child were together in the same space before a child was lost. If accompanied, the second prerequisite was the physical distance between the guardian and the child, that is, whether the guardian was within the “control distance” that could prevent the child from leaving. In this study, the control distance was established when the child and guardian held hands or were right next to each other but not holding hands, allowing the guardian to immediately restrain the child’s movement. Additionally, even if the child was within the guardian’s range of sight, if the child went out of the control distance, the guardian’s likelihood of restraining the child from leaving might be lowered. In this context, both cases where children were within the guardian’s range of sight and were not accompanied by a guardian were considered to be outside the guardian’s control distance. These varying conditions are illustrated in Figure 2.

The sequence data for analyzing the sequential association rule on the occurrence of lost children was investigated by examining each lost child case and arranging them in order based on the conditions (situations) and causes affecting the occurrence. The presence or absence of accompaniment was classified as [A.O] and [A.X], respectively, and the presence or absence within the control distance was classified as [C.O] and [C.X], respectively. However, even if the guardians were within the control distance, there were cases where children were lost on transportation, such as elevators, subways, and buses. In this particular case, instead of [C.O] and [C.X], [T.O] and [T.X] represented the presence or absence of a control distance, respectively, within the boarding space.

This study used the causes of lost children, as shown in Table 1, which were suggested in the previous study. “G1–G4” were causes by guardians, “C1–C8” by children, and “E1–E8” by the environment. Among the causes by children, “Other–Child moves away” was coded as “C@” since it includes cases in which the children got lost by moving away for unknown reasons.

ECMiner, a data mining analysis program, was used for the analysis [36]. During the analysis, the minimum support and confidence values as well as the sequence pattern lengths were set as follows. ECMiner was utilized to analyze the number of supports when establishing the minimum support. In this study, only two or more rules were considered. In addition, to derive a rule with an accuracy of more than half, the minimum confidence was established at 50%. Additionally, the length of the pattern (the number of items) that determines the maximum sequence length was set to six. This reflected that the maximum pattern length of the cause combinations with two or more frequencies was four. In other words, the total pattern length was set to six by adding each condition (situation) of “the presence or absence of accompanying guardian and child”, “presence or absence of guardian’s child control distance”, and the maximum pattern length of the cause combination.

### 3.2. Derivation of the Rules That Cause Lost Children Using the Sequential Association Rule

**Figure 3:**
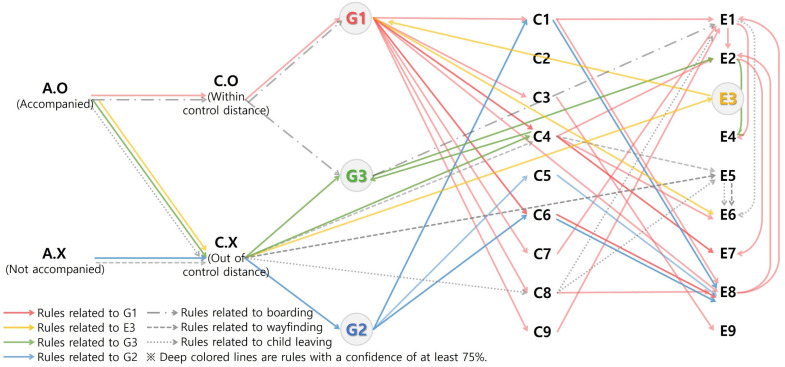
Visualizing the sequential association rules of lost children.

Based on the analysis criteria established earlier, the sequential association rules for the 202 cases of lost children were analyzed, and as a result, a total of 137 rules were derived. Since these rules corresponded to frequent sequences (item sets) that commonly occur, it was required to identify the maximum sequence rule, excluding the rules with partially overlapping items. Accordingly, when a rule with a short pattern length (fewer connected items) was included within a rule with a long pattern length (numerous connected items), the maximum sequence rule was selected by excluding the rule with a short pattern length. For instance, as the rule “[C5]---->[E8]” was part of the rule “[AX]-->[CX]-->[G2]-->[C5]-->[E8]”, it was excluded from the final rule. Moreover, among the selected rules, the rule identifying a coincidence as a lift value of 1 or a negative correlation as a lift value of less than 1 was excluded. Using this method, 27 out of 137 rules were derived (Table 2). The 27 sequential association rules were visualized, as shown in Figure 3.

The derived rules indicate a pattern connected in the order of whether the guardians and children were accompanied, the control distance, and the cause. The cause that predominantly appeared was the negligence of the guardians (G1–3), which corresponded to 21 (77.8%) of the 27 rules. It generally appeared as a pattern of causing a lost child situation through the child leaving while the guardian was negligent, and the environmental characteristics made it difficult for the guardian to find the child and for the child to find his or her way. Alternatively, patterns leading to the occurrence of lost children also appeared (22.2%), with a preceding cause other than the guardian, due to a specific situation. For example, there were cases where, due to the environmental characteristics (E3) that preceded the guardians’ negligence, the phenomenon of children leaving their seats occurred. Additionally, children were also lost by them suddenly leaving during playtime (C8), wayfinding failure due to the environment (E5, E6), or sudden movement (operation) of transportation, etc. This means that the patterns and processes of a child leaving may also vary depending on the cause or situation that formed the starting point of the lost child. Therefore, when classifying the rules that resulted in lost children, this study attempted to classify and categorize them based on the cause or situation that became the starting point.

## 4. Classification of the Types and Processes of Lost Children

### 4.1. Types of Lost Children

The characteristics of child loss were typified based on the previously classified 27 rules, and the processes of lost children were systematized using the type. The characteristics of child loss were considered from the perspectives of “behavior” and “situation” rather than the “cause” of the guardians and children. To derive the types of lost children, the operational definition of “child loss” was considered to be “a child’s physical separation from the guardian without the recognition of the guardian and the child”.

**Figure 4:**
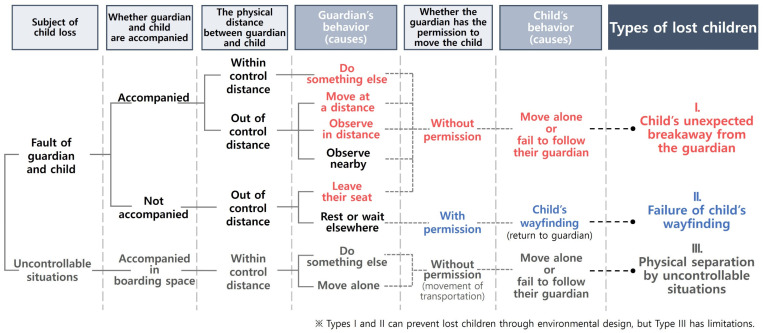
Criteria for typifying lost children.

The types of lost children were derived into three types (seven detailed types), and the derivation process is shown in Table 3 and Figure 4. The criteria for the classification of the types were as follows. The type of lost child was classified according to the “subject of child loss”. In other words, lost child occurrence was classified by whether a child got lost due to the “guardian’s or child’s fault” or “another’s fault or an uncontrollable situation”. When it was due to the “guardian’s or child’s fault”, the occurrence was classified by whether the guardian accompanied the child. It was further classified by the physical distance between the guardian and child when child loss occurred while the child was accompanied by the guardian. Additionally, it was classified by whether the child was within the guardian’s control range and the behaviors of the guardian in such instances, i.e., whether the guardian was doing something else, whether the guardian left the child for something else, and whether the guardian and child kept their distance while walking. When a child’s breakaway occurred due to the guardian’s behavior, the occurrence of a lost child was categorized based on whether the breakaway took place with the guardian’s permission. The movement of a child after obtaining the guardian’s permission could not be regarded as a situation of child loss. However, movement without permission was an unexpected breakaway. A lost child occurrence due to the child’s sudden breakaway (I) was a case where a child got lost by accidentally moving away even though the guardian and child were together. Another type (II) of a lost child occurrence due to the guardian’s or child’s fault was when a child tried to get back to the guardian and failed with wayfinding. The other type (III) referred to uncontrollable child loss from physical separation due to the operation of automated transportation, such as elevators, subways, buses, etc. To sum up, types I and II are distinguished from type III by the “subject of child loss”, and types I and II are distinguished by whether the guardian accompanied the child. Type I, which corresponds to most cases of lost children, was further classified based on the guardians’ behavior, which was the starting point of child loss. The guardian’s behavior leading to child loss could mainly be classified as “doing something else”, “walking by oneself”, “leaving their seat”, and “observing the child from a distance”. Type I-1 refers to when the child moves while the guardian is doing something else, type I-2 refers to when the guardian and child walk separately (thinking the other will follow), type I-3 refers to when the child moves while the guardian has left their seat, type I-4 refers to when the child moves while the guardian is observing from a distance, and type I-5 refers to when the child suddenly moves even though the guardian is observing.

### 4.2. Process of Lost Children by Types

**Figure 5:**
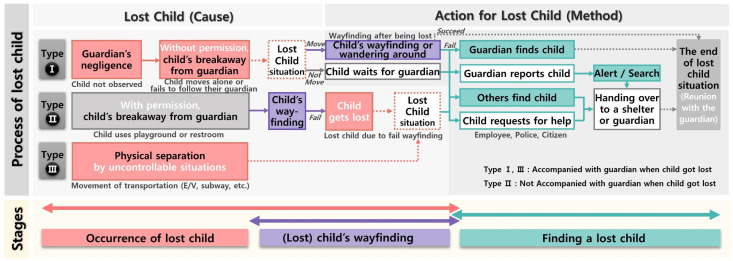
Process of lost children by types.

The process of lost children can be divided into three stages: “occurrence of a lost child”, “wayfinding of a lost child”, and “finding a lost child” (Figure 5). The standard time of occurrence of a lost child can be regarded as the moment when the child leaves without the guardian’s perception while the guardian accompanies the child, and the lost child attempts to find their way or wander around to find their guardian. Alternatively, if the child is not accompanied by a guardian (when the child leaves with the guardian’s permission), it can be considered that the child is lost from the moment they lose their way and cannot find it alone. A lost child can overcome this situation by asking for assistance from people around them or by being found. Additionally, the guardian may recognize that the child has disappeared and find the child by reporting them to the police or the facility’s staff and searching for the lost child.

This series of the lost children processes is summarized in detail by type as follows.

Type I represents the most common type of lost child. In this type, the child suddenly abandons the guardian due to various reasons such as the guardian’s negligence, the child’s activeness and curiosity, or environmental characteristics in diverse places. The child becomes lost by moving alone without the guardian’s recognition or by failing to catch up with the guardian. In these situations, children react in three ways. They stay in place, wander to search for their guardian, or ask for help. The probability of quickly finding the child increases if the child stays in place and waits for the guardian. If the child wanders to search for the guardian and succeeds with wayfinding, he or she can reunite with the guardian. However, if the child fails with wayfinding, the child may be farther away from the guardian, making it even more difficult to reunite. If the guardian also wanders to search for the child in this situation, it may take even more time to find each other. Such situations may be overcome when the child is discovered by others or asks for help.

Type II is when a child leaves for the playground or restroom after obtaining the guardian’s permission. Both the guardian and child are aware of their physical breakaway, and the child is not in a lost condition yet. The child is not lost if they find their way back. However, if the child fails with wayfinding while trying to get back to the guardian, the child becomes lost. The child may be discovered by others or ask for assistance to overcome this situation.

Type III is when a child gets lost not due to the guardian’s or child’s fault but to uncontrollable situations. While using transportation, such as an elevator, subway, or bus, the guardian and child may be accidentally separated by a sudden door closure. The situation may be overcome by the guardian going back to the child or other people assisting the child.

All three types involve a process of a child getting lost and overcoming the situation. However, type III is exceptional in that the child gets lost in an uncontrollable situation. Types I and II are similar in that it is the guardian’s or child’s fault. However, type I is under the condition that the guardian is accompanying the child, while type II is of them being unaccompanied and physically separated.

## 5. Analysis of the Characteristics of Lost Children by Types

### 5.1. Classification of the Causes and Space in Each Type of Lost Child

In this section, 202 lost child cases were classified by type, and the causes of each type were systemically categorized and analyzed for their characteristics. Additionally, the causes of each type were statistically compiled and proportioned. Based on the frequency (the number of cases in which lost children occurred due to the cause in each type) and proportion (the ratio of the number of cases in which lost children occurred due to the cause in the number of cases by type), the relationship between each type and its cause were identified.

When determining the degree of association between the causes of lost children by type, the “case ratio” and “confidence” of the lost children occurrence rule derived earlier were used as the standard indicators. In other words, the degree of each cause’s representativeness in the corresponding type was judged as the case ratio by cause. The validity of each cause included in the corresponding type was assessed as the confidence index of the rule. Furthermore, the criteria for determining the association were set as follows. The criterion for the confidence was based on 50%, which comprised a majority of the lost children occurrence rule. The case ratio by cause was established based on the probability that the cause affected the occurrence of lost children in the cause’s category (guardian, child, and environment) for each type. For example, in the case of type I-1, the average number of causes affected per case in each cause category was 1.05 by guardians, 0.94 by children, and 1.16 by environments. Moreover, the number of guardians, children, and environmental causes was three, nine, and nine, respectively. The probability of losing a child due to each cause in the corresponding type was 35.0% for the guardian cause category, 10.4% for the children cause category, and 12.9% for the environmental cause category. A value higher than this criterion indicated that the cause had a greater influence on the incidence of lost children than the average, and a lower ratio value indicated that it had a lower impact on the incidence of lost children. Furthermore, if the cause of each category exceeded the standard ratio in the type, the cause was judged as closely related to the type (Table 4).

The degree of association between the causes of lost children by type was classified and marked as “strong, medium, weak, or nothing”. In addition, since the lost children occurrence rule only reflected the rules with two or more frequencies (support), the confidence concerning the cause of the cases with one frequency was not derived. Thus, if the cause of the cases with one frequency inferred that there was a possibility of causing a lost child in the corresponding type, it was separately marked with “(○)”. Similarly, when determining the association of the functional spaces, the probability of 12.5% for causing lost children in eight functional spaces was used as the standard. The process of causing lost children in each space by type was examined and comprehensively determined (Table 5 and Table 6).

### 5.2. Causes and Characteristics of Lost Children by Types

#### 5.2.1. Type I: Child’s Unexpected Breakaway from the Guardian

1. Type I-1: Child moving while guardian is doing something else.

Type I-1 occurs when a guardian is doing something else without recognizing the danger of the child getting lost because the child is within the control range. This is the most common type of lost child and may occur in any kind of functional space. The reasons why guardians did not observe children within a functional space are as follows. In sales spaces, parents were unable to observe the children while looking at the products, purchasing, or inquiring with the staff. In amusement and rest spaces, children playing were not observed as their guardians cared for other children or talked to acquaintances, used cell phones, or ate. In food and beverage (F&B) spaces, guardians did not pay attention to their children as they ordered and paid for food, cleaned up after eating, or were busy eating. In convenience spaces, guardians did not pay attention to children while washing their hands in the bathroom, inquiring with staff, or organizing luggage in customer centers/stroller rental offices. In circulation spaces, guardians could not keep an eye on their children as they were looking at products or interesting factors, caring for other children, looking for items, organizing luggage, or talking. In watching spaces, guardians and children did not pay attention to each other as they viewed the exhibits. A child moves for various reasons, especially regarding interesting factors or for playing, which results in them moving out of the control range and getting lost. Children are more likely to get lost in environments with many visual obstacles, a high density of people, or open structures without any borders.

2. Type I-2: Child and guardian walking separately.

Type I-2 occurs when the guardian moves alone, assuming that child is following them or finding his/her way in a situation where the guardian and child are within the visible range of the same space but outside the control distance. The reason for the guardian and the child moving separately was mainly that the guardian moved without holding the child’s hand to carry the luggage with both hands or to hold the hand of a younger child. In addition, there were several cases where the guardians and children moved separately from frequently visited familiar places, such as near their homes or kindergartens. The child may be distracted by interesting factors, move unconsciously, or follow someone else who looks similar to the guardian. Alternatively, the child may assume that the guardian would follow them and move ahead alone. This type is based on the guardian’s trust and the child’s independence. Therefore, it occurs to children around the age of 4.2 years on average, which is relatively higher than 3.6 years, which is the average age of type I-1 (representative lost child type). Such situations occur in an environment with a high density of people, many visual obstacles, or structural complexity since the guardian and child move separately. This type mainly occurs in circulation spaces.

3. Type I-3: Child walking away while the guardian leaves their seat.

In type I-3, the guardian and child become physically separated when the guardian leaves and the child becomes lost by moving alone without waiting for the guardian or leaving for interesting factors. This type occurs in children aged 4.1 years on average, which refers to preschool children rather than infants. This type may occur in most spaces except for circulation and boarding spaces. In the amusement space, there were cases where the guardians who were observing the children playing were absent for a while to use the bathroom or go shopping. In the sales space, the children were waiting in front of the ticket office or in the store, and the guardian left to pay, inquire, or use the bathroom. In the rest/reading space, while the children were resting or reading on the bench, the guardians left their seats to use the bathroom or go shopping. In the F&B space, there were cases where the guardian went to order food, pick up food, or return dishes while the child was sitting at a table and waiting. As such, if the child does not leave when the guardian is away, the guardian and child are reunited again, but if the child leaves without waiting for the guardian, the child may become lost. The most common reason for children to leave was to find their guardians, and in some cases, they left after seeing interesting factors or following someone wearing clothes similar to their guardian by mistaking them for their guardian. It is easier for a child to get lost in an environment with an open structure, a high density of people, or many visual obstacles.

4. Type I-4: Child walking away while the guardian observes from a distance

Dissimilar to the previous types, type I-4 results in lost children preceded by environmental characteristics rather than guardian causes, and mainly occurs in amusement spaces. When the guardian’s area is scarce or located far from the child’s area, the child becomes lost since the guardian inevitably observes the child from a distance. The child becomes lost outside the control range but within the visible range while the guardian is doing something else or leaves the area. When the guardian is unable to watch over the child, the child may leave to play or follow interesting factors. Since this type refers to a guardian supervising the child from a distance, it occurs in an environment with visibility constraints, such as a high density of people, many visual obstacles, or an open structure that causes children to leave easily. If the guardian’s area lacks design differentiation or is located in a complex structure, the child may find it difficult to return to the guardian. The most fundamental cause of this type may involve the guardian’s space being designed in such a way that it makes the child’s observation difficult.

5. Type I-5: Child suddenly running

Type I-5 occurs when the guardian and child are in the same space, but the child suddenly leaves and becomes lost even though the guardian is not careless. Most children become lost when their guardians do not hold their hand. However, even if they hold hands, the child may suddenly release the guardian’s hand and leave. This type mainly occurs in watching and amusement spaces. In watching spaces, when the guardian and child were watching something together, the child quickly left the guardian to see the exhibits or animals and became lost. In amusement spaces, there were cases where children suddenly ran at a high speed to play or left without their guardian’s knowledge to play hide-and-seek. At this time, if the guardian observes and controls the movement of the child, the child does not become lost. However, if the guardian does not see the child while doing something else, the child becomes lost. Additionally, although the guardian is aware of the child’s breakaway, they may visually miss the child’s movement as the child is concealed by crowds or visual obstacles.

#### 5.2.2. Type II: Failure of Child’s Wayfinding

Type II occurs when a child gets lost on the way back after separation under the guardian’s permission. This type is based on the guardian’s trust in the child’s independence, similar to type I-2, and the average age of children in this type is 5.9 years, which is relatively high. It usually occurs in amusement, circulation, and F&B spaces. In the amusement space, the guardian’s resting space is insufficient or far from the amusement space, so the guardian rests from a distance, and the child moves to the amusement space alone to play. In the F&B space, the restroom is far away; thus, while the guardian eats, the child moves to the restroom alone. Afterward, children who have left with the guardian’s permission attempt to return to their guardians by utilizing the sign system and spatial memory. However, on the way back to the guardian, the child may fail with wayfinding due to the complexity of the structure, lack of architectural differentiation, or unclear sign system. In this type, environmental causes have more influence on the occurrence of lost children than the guardians or children, and children are more likely to fail in finding their way when they visit a place for the first time.

#### 5.2.3. Type III: Physical Separation by Uncontrollable Situations

Type III is an unusual case that occurs in boarding spaces where the space changes physically. This is when a guardian and child are accidentally separated by a sudden operation of transportation, such as elevators, subways, or buses. This type occurs when the guardian does not hold the child’s hand to carry something or holds another younger child’s hand, although the child is within their control distance. Such situations usually occur in high-density spaces or familiar places such as residential elevators. This type is similar to types I-1 or I-2 in that it occurs when the guardian does something else or moves first. However, there is a difference in that, due to the guardian’s and child’s space being physically separated and moved, they cannot return directly to each other. In addition, it can be seen as a force majeure in that the guardian cannot control the physical separation of the transportation means, such as a door closure. In other words, since the space is physically separated and moving, there were many cases where the lost child was found by passersby rather than the guardian looking for the child.

## 6. Conclusions

This was a fundamental study for preventing lost children, and the types of lost children were derived by implementing the sequential association rule based on the existing lost child cases. A total of 137 association rules were derived, and the types of lost children were derived by grouping these rules into types with common attributes. In addition, the process of children being lost and reuniting with their guardians was systematized by type. The types of lost children were categorized into three main categories and seven detailed categories. The most common type was type Ⅰ, which occurs when a child unexpectedly walks away from the guardian. Type I can be classified into detailed types according to the behavior of the guardian, which is the starting point for the occurrence of a lost child. The detailed types included the type in which the child moves and becomes lost while the guardian is doing something else (I-1), the guardian and child move separately (I-2), the child walks away while the guardian leaves their seat (I-3), the child walks away while the guardian observes from a distance (I-4), and the child suddenly runs (I-5). Type II, which mostly occurs in children of relatively older ages, was when a child leaves after obtaining the guardian’s permission but fails with wayfinding on the way back to the guardian. The last type, type III, was unique and refers to when a guardian and child are separated by transportation, such as an elevator, a subway, or a bus. Each type of lost child differs in both the main cause and functional space where it tends to occur. In other words, each functional space has different types of lost children that may be predicted to occur. Therefore, controlling the environment according to the characteristics of each type will lead to preventing children from getting lost.

The research on deriving the type and process of lost children that was conducted in this study has rarely been conducted in the past, including in the fields of both children and architecture. In other words, this study is significant as it systematically establishes the basic research related to lost children. Accordingly, the contents of this study can be used as the basic theoretical materials for future studies related to lost children in various fields.

Notwithstanding the many strengths of this study, it also contains some limitations. For instance, the causes and types of lost children were derived using a case study of lost children based on interviews with guardians. In other words, the types of lost children were classified from the perspective of the guardian, i.e., how the guardian lost and found their child. The case study lacked an approach from the child’s perspective, such as how they experienced getting lost and how they tried to find their way back. Consequently, this study classified the type of lost child due to the child’s unexpected breakaway from the guardian into five detailed types. However, the type of lost child due to failure with their wayfinding was collectively referred to as one type. There are various patterns that influence the success and failure of children’s wayfinding, with various environmental factors acting in the process. Therefore, in order to suggest environmental planning techniques to facilitate children’s wayfinding and prevent them from getting lost, additional research is needed on the characteristics of wayfinding and the causes from the child’s perspective. In the future, these limitations should be addressed by conducting experimental studies on children’s wayfinding methods.

## Figures and Tables

**Table 1:** Causes of lost children [16].

**Guardian**	G1	Guardian is doing something else.
	G2	Guardian leaves the child.
	G3	Guardian moves away, thinking that the child is following the guardian, or the child is able to find his/her way.
	G4	Guardian does not care about the child, thinking that the other guardian was taking care of the child.
**Child**	C1	Child spontaneously moves toward the interesting factors.
	C2	Child recognizes and moves toward the interesting factor with past memories.
	C3	Child moves to a place by himself/herself before the guardian moves (egocentrism).
	C4	Child moves alone, thinking that the guardian would come right after him/her (independence).
	C5	Child tries to find the guardian even though the guardian told the child to wait.
	C6	Child follows a person who looks or dresses like the guardian.
	C7	Child moves in the direction of the people or straight ahead.
	C8	Child moves away from the guardian to play or fool around.
	C@	Other—Child moves away/is unable to follow the guardian (unclear reason).
**Environment**	E1	Child is blocked and pushed by a high density of people.
	E2	Child is covered by visual obstacles.
	E3	Guardian watches over the child from far away because the guardian’s space is insufficient or too far away from the child’s space.
	E4	Child searches for the guardian because the guardian is covered by visual obstacles or a crowd.
	E5	Child is not able to return due to the complexity of a structure.
	E6	Child is not able to return due to lack of architectural differentiation.
	E7	Child is not able to return due to many visual obstacles that are higher than the child’s height.
	E8	There is an open structure without a door/fence (border) that is easy for the child to leave alone.
	E9	Guardian is not able to properly watch the child because the surroundings are dark.

**Table 2:** Deriving and classifying the rule (pattern) of lost children using the sequential association rule.

Rules	Sequential Association Rules for the Occurrence of Lost Children (27)		Connection	Confidence (%)	Lift	Support (%)
**Related to G1**	1	[A.O]-->[C.O]-->[G1]-->[C1]-->[E1]---->[E2]	6	50.0	3.1	1.5
	2	[A.O]-->[C.O]-->[G1]-->[C1]-->[E8]---->[E2]	6	50.0	3.1	1.5
	3	[A.O]-->[C.O]-->[G1]-->[C3]---->[E9]	5	50.0	9.2	1.0
	4	[A.O]-->[C.O]-->[G1]-->[C4]---->[E2]	5	66.7	4.1	1.0
	5	[A.O]-->[C.O]-->[G1]-->[C4]---->[E6]	5	66.7	5.2	1.0
	6	[A.O]-->[C.O]-->[G1]-->[C4]---->[E7]	5	100.0	28.9	1.0
	7	[A.O]-->[C.O]-->[G1]-->[C6]---->[E8]	5	100.0	5.1	1.0
	8	[A.O]-->[C.O]-->[G1]-->[C7]---->[E1]	5	50.0	2.0	2.0
	9	[A.O]-->[C.O]-->[G1]-->[C8]-->[E8]---->[E2]	6	66.7	4.1	1.0
	10	[A.O]-->[C.O]-->[G1]-->[C@]-->[E8]---->[E6]	6	60.0	4.7	1.5
	11	[A.O]-->[C.O]-->[G1]-->[E8]-->[E1]---->[E4]	6	50.0	5.1	1.0
	12	[A.O]-->[C.O]-->[G1]-->[E8]-->[E2]---->[E7]	6	50.0	14.4	2.0
	13	[A.O]-->[G1]-->[C9]---->[E1]	4	50.0	2.0	1.0
**Related to G3**	14	[A.O]-->[C.X]-->[C4]---->[G3]	4	88.9	4.3	4.0
	15	[A.O]-->[G3]-->[E2]---->[E4]	4	100.0	10.1	1.0
	16	[C.X]-->[G3]-->[E2]---->[E4]	4	100.0	10.1	1.0
**Related to G2**	17	[A.X]-->[C.X]-->[G2]-->[C1]---->[E8]	5	75.0	3.8	1.5
	18	[A.X]-->[C.X]-->[G2]-->[C5]---->[E8]	5	50.0	2.5	1.5
	19	[A.X]-->[C.X]-->[G2]-->[C6]---->[E8]	5	100.0	5.1	1.0
	20	[A.X]-->[C.X]-->[G2]-->[C@]---->[E8]	5	50.0	2.5	1.0
**Related to E3**	21	[A.O]-->[C.X]-->[E3]-->[G1]---->[E6]	5	100.0	7.8	1.0
**Related to child leaving suddenly**	22	[A.O]-->[C.X]-->[C8]-->[E1]---->[E6]	5	66.7	5.2	1.0
	23	[A.O]-->[C8]-->[E5]---->[E6]	4	50.0	3.9	1.0
**Related to wayfinding**	24	[A.X]-->[C.X]-->[E5]---->[E6]	4	100.0	7.8	1.5
	25	[C.X]-->[C4]-->[E5]---->[E6]	4	50.0	3.9	1.0
**Related to boarding**	26	[A.O]-->[T.O]-->[G1]---->[C3]	4	66.7	15.0	1.0
	27	[A.O]-->[T.O]-->[G3]---->[E1]	4	66.7	2.7	1.0

**Table 3:** Deriving the types of lost children.

Accompaniedor Not	Physical Distance	Guardian’s Behavior (Causes)	Child’s Behavior (Causes)	Environmental Characteristics (Causes)	Types of Lost Children		
Accompanied	Within control distance	(G1)	(C1), (C3), (C4), (C6), (C7), (C8), (C9)	(E1), (E2), (E4), (E6), (E7), (E8), (E9)	**Ⅰ-1.** **Child moving while guardian is doing something else**	**Ⅰ. Child’s unexpected breakaway from the guardian**	**Fault of guardians and children**
	Out of control distance	(G1), Guardian observes children from a distance (–)	Child moves to guardian (–)	(E3), (E6)	**Ⅰ-4.** **Child walking away while the guardian observes from a distance**		
		(G3)	(C4)	(E2), (E4)	**Ⅰ-2.** **Child and guardian walking separately**		
		Guardian observes children nearby (–)	(C8)	(E1), (E5), (E6)	**Ⅰ-5.** **Child suddenly running**		
Not accompanied	Out of control distance	(G2)	(C1), (C5), (C6)	(E8)	**Ⅰ-3.** **Child walking away while the guardian leaves their seat**		
		Guardian rests or waits elsewhere (–)	(C4), Child’s way finding (–)	(E5), (E6)	**Ⅱ.** **Failure of child’s way finding**		
Accompanied in boarding space	Within control distance	(G1)	(C3)	Movement of transportation (–)	**Ⅲ.** **Physical separation by uncontrollable situations**		
		(G3)	Child fails to follow their guardian (–)	(E1), Movement of transportation (–)			

Notes: Not all behavior causes lost children. This table shows the behavior of guardians and children at the time of the occurrence of a lost child. (–) means that neither the guardians nor the children were at fault.

**Table 4:** Criteria for determining the association of the lost child causes by type (case ratio).

Type	Cause Category	Guardian	Child	Environment
Type Ⅰ-1	Average number of causes	1.05	0.94	1.16
	Criterion for case ratio	35.0%	10.4%	12.9%
Type Ⅰ-2	Average number of causes	1.25	0.89	0.58
	Criterion for case ratio	41.7%	9.9%	6.4%
Type Ⅰ-3	Average number of causes	0.96	0.87	0.78
	Criterion for case ratio	32.0%	9.7%	8.7%
Type Ⅰ-4	Average number of causes	0.25	0.50	2.13
	Criterion for case ratio	8.3%	5.6%	23.7%
Type 1-5	Average number of causes	0.09	1.00	1.64
	Criterion for case ratio	3.0%	11.1%	18.2%
Type Ⅱ	Average number of causes	0.14	0.43	1.29
	Criterion for case ratio	4.7%	4.8%	14.3%
Type Ⅲ	Average number of causes	1.00	0.63	0.25
	Criterion for case ratio	33.3%	7.0%	2.8%

**Table 5:** Degree of association.

By Type of Lost Children		Case Ratio	
		Above Criteria for Case Ratio	Below Criteria for Case Ratio
Confidence of rules for the occurrence of lost children	Above 50%	● (Strong)	◐ (Medium)
	Below 50%	◐ (Medium)	○ (Weak)

Notes: (○)—Among the causes with few cases, it was inferred that there was a possibility of causing a lost child. The G4 cause was also judged by the inference as a secondary cause, not the main cause.

**Table 6:** The causes and characteristics of lost children by types.

Types of Lost Children			I											II		III	
			Child’s Unexpected Breakaway from the Guardian											Failure of Child’s Wayfinding		Physical Separation by Uncontrollable Situations	
			I-1		I-2		I-3		I-4			I-5					
**202 cases of lost children**			109 (54.0%)		36 (17.8%)		23 (11.4%)		8 (4.0%)			11 (5.4%)		7 (3.5%)		8 (4.0%)	
**Average age of lost children**			3.9											5.9		2.3	
			3.6		4.2		4.1		4.1			4.8					
**Physical distance between guardian and child**		**Accompanied** **(Same space)**	●		●		x		●			●		x		●	
		**Within controllable distance**	●		●		x		x			●		x		●	
**Guardian’s permission**			x		x		x		x			x		●		x	
**Causes of lost children**	**Guardian**	**G1**	●	107 (98%)	◐	4 (11%)	-	0	●	2 (25%)		○	1 (9%)	-	0	●	3 (38%)
		**G2**	-	0	-	0	●	22 (96%)	-	0		-	0	-	0	-	1 (13%)
		**G3**	-	1 (1%)	●	36 (100%)	-	0	-	0		-	0	(○)	1 (14%)	●	4 (50%)
		**G4**	(○)	6 (6%)	(○)	5 (14%)	(○)	4 (17%)	-	0		-	0	-	0	-	0
	**Child**	**C1**	●	24 (22%)	◐	9 (25%)	●	4 (17%)	-	0		◐	6 (55%)	-	0	-	0
		**C2**	(○)	10 (9%)	-	0	-	0	-	0		-	0	-	0	-	0
		**C3**	◐	5 (5%)	-	0	-	1 (4%)	-	0		-	0	-	0	●	3 (38%)
		**C4**	◐	4 (4%)	●	8 (22%)	-	0	-	0		(○)	1 (9%)	●	1 (14%)	-	0
		**C5**	-	0	-	0	●	7 (30%)	(○)	1 (13%)		-	0	-	0	-	0
		**C6**	◐	2 (2%)	○	3 (8%)	◐	2 (9%)	(○)	1 (13%)		-	0	-	0	-	0
		**C7**	◐	8 (7%)	◐	5 (14%)	-	1 (4%)	-	0		-	0	-	0	(○)	1 (13%)
		**C8**	●	13 (12%)	(○)	1 (3%)	-	0	◐	2 (25%)		●	4 (36%)	(○)	2 (29%)	(○)	1 (13%)
		**C@**	◐	4 (4%)	(○)	1 (3%)	-	1 (4%)	-	0		-	0	-	0	-	0
	**Environment**	**E1**	●	27 (25%)	◐	7 (19%)	◐	3 (13%)	◐	3 (38%)		●	7 (64%)	(○)	1 (14%)	●	2 (25%)
		**E2**	●	25 (23%)	●	3 (8%)	◐	2 (9%)	(○)	1 (13%)		○	2 (18%)	-	0	-	0
		**E3**	-	0	-	0	-	0	●	6 (75%)		-	0	(○)	1 (14%)	-	0
		**E4**	●	15 (14%)	●	5 (14%)	-	0	-	0		-	0	-	0	-	0
		**E5**	(○)	7 (6%)	◐	4 (11%)	(○)	1 (4%)	(○)	1 (13%)		●	2 (18%)	●	2 (29%)	-	0
		**E6**	◐	10 (9%)	○	1 (3%)	(○)	1 (4%)	●	4 (50%)		●	5 (45%)	●	5 (71%)	-	0
		**E7**	◐	7 (6%)	-	0	-	0	-	0		-	0	-	0	-	0
		**E8**	●	29 (27%)	-	0	●	10 (43%)	(○)	1 (13%)		-	0	-	0	-	0
		**E9**	◐	6 (6%)	(○)	1 (3%)	-	1 (4%)	(○)	1 (13%)		(○)	2 (18%)	-	0	-	0
**Functional space**		**Sales**	●	50 (46%)	○	4 (11%)	●	4 (17%)	-	0		○	1 (9%)	-	0	-	0
		**Circulation**	●	15 (14%)	●	26 (72%)	○	2 (9%)	-	0		○	2 (18%)	●	2 (29%)	-	0
		**Amusement**	●	15 (14%)	-	0	●	7 (30%)	●	8 (100%)		●	3 (27%)	●	3 (43%)	-	0
		**Rest**	○	10 (9%)	○	2 (6%)	●	4 (17%)	-	0		-	0	-	0	-	0
		**Food and Beverage**	○	9 (8%)	-	0	●	4 (17%)	-	0		-	0	○	1 (14%)	-	0
		**Watching**	○	3 (3%)	○	1 (3%)	○	2 (9%)	-	0		●	5 (45%)	○	1 (14%)	-	0
		**Convenience**	○	7 (6%)	-	0	-	0	-	0		-	0	-	0	-	0
		**Boarding**	-	0	○	3 (8%)	-	0	-	0		-	0	-	0	●	8 (100%)

Notes: Frequency—the number of lost child cases due to the cause or functional space in each type. Percentage—the percentage of the number of lost child cases due to the cause or functional space out of all the cases of each type. Association criteria—strong “●”, medium “◐”, weak “○”, weak (inference) “(○)”, none “-”; ref. Table 4 and Table 5.

## Data Availability

The data presented in this study are available on request from the author.
